# Management of Severe Hypothermia: Challenges and Advanced Strategies

**DOI:** 10.3390/jcm14051584

**Published:** 2025-02-26

**Authors:** Bogdan Oprita, Ionut Olaru, Liviu Botezatu, Alice Elena Diaconu, Ruxandra Oprita

**Affiliations:** 1 Faculty of Medicine, University of Medicine and Pharmacy “Carol Davila”, 4192910 Bucharest, Romania; bogdan.oprita@umfcd.ro (B.O.);; 2 Emergency Department, Clinical Emergency Hospital of Bucharest, 050463 Bucharest, Romania

**Keywords:** severe hypothermia, IVTM, UPU-SMURD, controlled rewarming, ECMO, emergency medicine

## Abstract

Severe hypothermia is a medical emergency that can be fatal if not promptly and effectively managed. **Background:** This case report examines a patient with severe hypothermia and describes the advanced approaches utilized in treatment, highlighting the challenges encountered and the clinical decisions that led to a favorable outcome. **Methods**: We report a case of an approximately 60-year-old adult, who was found unconscious in low-temperature conditions, presenting with bradycardia and a measured core temperature of 25 °C. **Results**: Medical intervention included active internal rewarming and the use of a controlled intravascular active heating system to support vital functions. **Conclusions:** This case underscores the importance of a multidisciplinary approach and the advantages of utilizing modern technologies in the management of severe hypothermia for selected cases.

## 1. Introduction

Severe hypothermia is defined as a core temperature drop below 28 °C and can lead to serious complications, including arrhythmias, respiratory failure, and even cardiac arrest. Despite advances in understanding the pathophysiology of hypothermia, managing this condition remains complex, requiring specialized equipment and a multidisciplinary team [1]. This report details the case of a patient with severe hypothermia and discusses the strategies employed to achieve a favorable outcome.

## 2. Case Report

The patient, I.S., is a 60-year-old male with unknown medical history of chronic illnesses or other health vulnerabilities. He is a homeless individual who was found outdoors in cold weather conditions (2 °C at night and a maximum of 9 °C during the day), dressed in wet clothes, and brought to the Emergency Department by ambulance.

On admission, the patient was confused and had an altered mental status, GCS of 11, and a core temperature measured rectally of 25 degrees centigrade.

The vital signs were as follows:

(a) Bradycardia: 38 bpm;
(b) Blood pressure: 160/74 mmHg;
(c) SpO_2_: 84% (corrected to 93% with oxygen administration via nasal cannula at 3 L/min);
(d) Normal, sluggishly reactive pupils;
(e) Glasgow Coma Scale (GCS) score of 11—altered level of consciousness.

Pulmonary auscultation revealed diminished vesicular breath sounds in the left lung base. Also, excoriated wound on the dorsal aspect of the nose was observed, likely caused by a fall or trauma during cold exposure.

Upon admission, the patient underwent a series of laboratory and imaging evaluations to assess the extent of hypothermia-related complications and other possible injuries.

**Figure 1:**
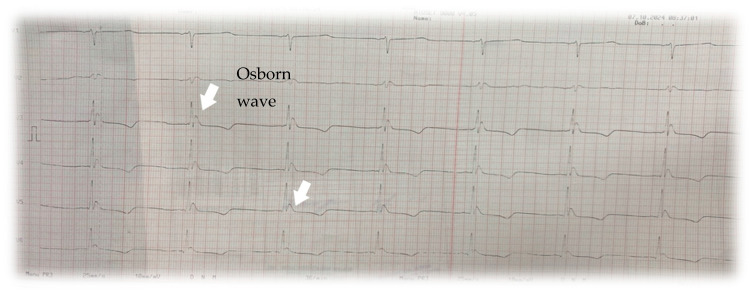
Electrocardiogram showing the Osborn wave, also known as “J wave” or “hypothermia wave”, a distinct positive deflection at the junction between the ORS complex and the ST segment secondary to heterogeneous repolarization between the epicardial and endocardial layers due to the differential response of myocardial cells to low temperatures [2].

➢ Pathological blood exam results are as follows:
  ○ pH: 7.31 (mild acidosis);
  ○ pH: 7.31 (mild acidosis);
  ○ Lactate: 5.8 mmol/L (hyperlactatemia);
  ○ Sodium (Na): 131 mmol/L (mild hyponatremia);
  ○ Potassium (K): 3.0 mmol/L (mild hypokalemia);
  ○ Glucose: 232 mg/dL (mild hyperglycemia).
➢ Electrocardiogram (ECG): Sinus bradycardia at 38 bpm and the presence of an Osborn wave (J wave) was revealed, a hallmark finding in hypothermia(Figure 1).
➢ Chest X-ray: No acute parenchymal pulmonary lesions were visible. Left-sided cardiac silhouette opacification and enlarged heart were observed.

Admission diagnosis for the presented patient was as follows: severe hypothermia; coma; craniofacial trauma with an excoriated wound on the dorsum nasi.

**Figure 2:**
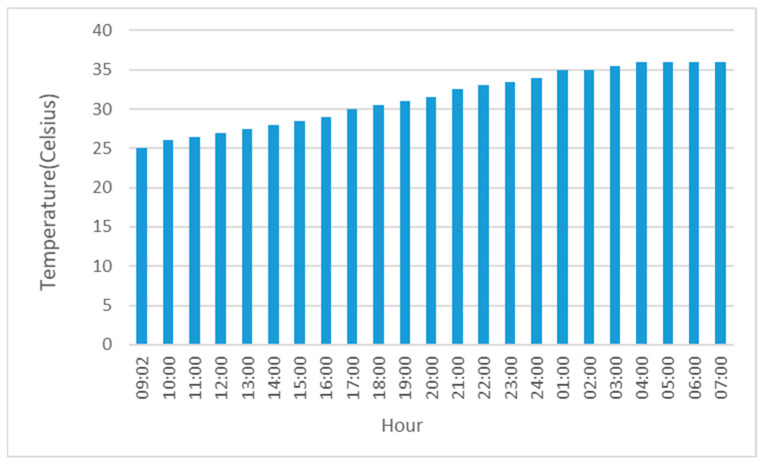
The graph depicts the evolution of core temperature during the rewarming process. A gradual and favorable response to rewarming is observed, with the target temperature being achieved at an approximate rate of 0.5–0.75 °C per hour, followed by the maintenance of a plateau at 35.7 °C during the final four hours of the rewarming phase.

Therapeutic management measures were implemented during the patient’s stay [3,4,5] in the emergency department, following the initial evaluation and placement in a warm environment, combined with the removal of wet clothing to minimize further heat loss; a protocol of both active and passive rewarming was initiated:

○ Passive Rewarming: The use of thermal blankets and placement of the patient in a warm environment.
○ Active Rewarming:
  (a) Administration of heated intravenous (IV) fluids: Intravenous saline solutions were warmed to a safe temperature of 41 °C using a temperature-controlled convection device. These fluids contributed to the gradual elevation of the patient’s core temperature and stabilization of vital signs.
  (b) Controlled intravascular temperature management (IVTM):

Given the patient’s sluggish response to the initial rewarming interventions during the first 2 h, an advanced invasive rewarming technique, IVTM [3], was employed to precisely regulate the rise in core temperature. This method, commonly used in intensive care settings, operates independently of the intravenous fluid volume administered. The system was programmed to increase the patient’s core temperature by 0.65 °C per hour until reaching 32 °C, and subsequently by 0.5 °C per hour until 35.7 °C (Figure 2).

Supportive interventions were initiated with continuous monitoring of vital signs, symptomatic treatment and supportive care for vital organ functions, correction of hydro-electrolytic imbalances and ongoing administration of warm intravenous fluids to maintain perfusion and homeostasis.

Approximately 120 min after initiating treatment, the patient’s core temperature rose from 25 °C to 26 °C, indicating modest improvement. Following the implementation of controlled intravascular rewarming (IVTM), the patient’s temperature increased steadily in accordance with the programmed settings, demonstrating progressive and sustained improvement.

About 14 h after admission to the Emergency Department, the patient’s core temperature reached 35 °C, with a heart rate of approximately 60 bpm, blood pressure of 145/90 mmHg, and a Glasgow Coma Scale (GCS) score of 13. However, psychomotor agitation required sedation with benzodiazepines. During this time, metabolic and acid–base imbalances were corrected, leading to normalization of pH, lactate, sodium, glucose, and potassium levels. Further diagnostic evaluations were performed during hospitalization, including computed tomography (CT) of the head, chest, abdomen, and pelvis with contrast, as well as echocardiography.

The patient’s initially stable clinical course took a turn for the worse with the development of bronchopneumonia. This necessitated multidisciplinary consultations (intensive care, cardiology, neurology, and psychiatry) for treatment recommendations. Despite the successful correction of hypothermia within the first 14 h, the patient succumbed to his condition 11 days after admission at the Clinical Emergency Hospital in Bucharest.

## 3. Discussion

Hypothermia refers to a state in which the body’s thermoregulatory mechanisms are overwhelmed due to prolonged exposure to low temperatures. It is defined as a reduction in core body temperature below 35.0 °C.

Common causes include the following: prolonged cold exposure, immersion or submersion accidents, comatose states (e.g., drug-induced, epileptic, metabolic), stroke, infections, endocrine insufficiency, extensive burns.

For establishing proper management, it is necessary to stage hypothermia. Determining the degree of hypothermia helps to stratify both immediate and long-term risks, guides resuscitation strategies based on body temperature, and ultimately informs treatment decisions. According to the study conducted by Marin E. Musi in 2021 [4], the most useful clinical staging scale for hypothermia is the Revised Swiss System, which classifies hypothermia as follows:

Stage 1: Core temperature between 35–32 °C—clear consciousness with shivering
Stage 2: Core temperature between 32–28 °C—impaired consciousness without shivering
Stage 3: Core temperature between 28–24 °C—unconsciousness
Stage 4: Core temperature between 24–14 °C—apparent death
Stage 5: Core temperature bellow 14 °C—death.

Additionally, the Wilderness Medical Society system divides hypothermia as follows:

1. Mild Hypothermia (32–35 °C), with symptoms like shivering, mild confusion, and weakness.
2. Moderate Hypothermia (28–32 °C), with symptoms including severe bradycardia, profound confusion, and impaired coordination.
3. Severe Hypothermia (<28 °C), a medical emergency with significant risks of cardiac arrhythmias, cardiovascular collapse, and death [4,6].

Another staging protocol is the Danish one, which is a simplified version of the Swiss system. The study also emphasizes that this staging has its limitations, especially in patients who present other causes of cognitive impairment.

**Figure 3:**
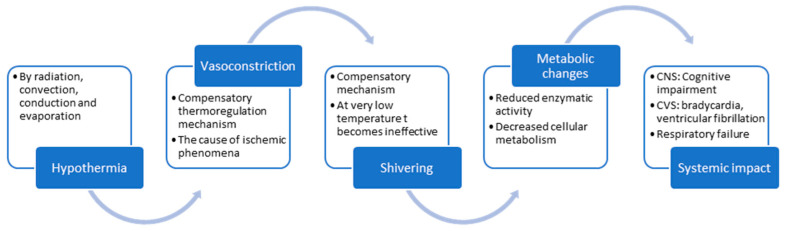
Schematic representation of the pathophysiological mechanism involved in hypothermia, beginning with the general triggering factor, progressing through immediate compensatory mechanism such as vasoconstriction and shivering, then through metabolic conservation and culminating in the systemic impact of hypothermia when the compensatory mechanisms have failed.

The pathophysiology of hypothermia (Figure 3) involves a multifaced interplay of metabolic suppression, cardiovascular compromise, neurological depression, hematological disturbance, renal impairment, and inflammatory responses. These processes are intricatly linked, with initial cellular dysfunction setting off a series of compensatory mechanisms that, while protective in the short term, ultimately contribute to systemic deterioration.

At the cellular level, the low temperature results in enzymatic inhibition and reduced ATP production. Ths energy deficit compromises essential processes such as ion transport and membrane integrity, forcing the installation of anaerobic metabolism and leading to the accumulation of lactate and metabolic acidosis. The cardiovascular system is affected by the decrease in myocardial contractility as a compensatory mechanism, but compounded by altered ion channel kinetics, which predisposes the heart to arrhythmias. In an attempt to preserve core temperature, the body induces peripheral vasoconstriction with the redistribution of blood flow. This will increase systemic vascular resistance and reduce cardiac output, which diminishes coronary perfusion and peripheral perfusion agravating the metabolic acidosis and myocardial ischemia. Also, the neurological system experiences a reduction in oxygenated blood leading to cerebral hypoperfusion, neuronal ischemia, cerebral edema, and, ultimately, persistent neurological deficits. The hematological system is affected by hypothermia which impairs platelet function and the enzymaic reactions within the coagulation cascade leading to disseminated intravascular coagulation (DIC). Renal function deteriorates as well, as hypothermia induces market renal vasococnstriction, predisposing the kidney to ischemic injury.

A critical challenge arises during the rewarming phase. The restoration of normothermia, while essential for recovery, can precipitate reperfusion injury and triggers rewarming shock—a state of hemodynamic instability that can lead to multi-organ failure.

For the treatment of severe hypothermia in a fully controlled manner, a comparative analysis was conducted to evaluate the benefits, complications, and costs of the method used (Intravascular Temperature Management, IVTM) against other advanced controlled warming methods found in the literature. Based on the search criteria, ECMO (Extracorporeal Membrane Oxygenation) was identified as the alternative therapeutic option. Below are the key features of both methods:

1. Controlled intravascular active warming method (used in this case):
  This is an advanced temperature management system designed to precisely and rapidly regulate the patient’s core temperature. It utilizes a specialized intravascular catheter with multiple heat-exchange balloons, through which cooled or warmed saline circulates within a closed circuit, without infusing fluid into the patient. As venous blood passes over the balloons, its temperature is adjusted, allowing for the efficient cooling or warming of the patient.
  The system comprises a console and a heat-exchange catheter with multiple balloons. Saline circulates through the catheter within the closed circuit, cooling or warming the patient as venous blood flows past the balloons, without the additional fluid infused in the circulatory system. The target temperature and rate of temperature adjustment are set, and the system modifies the saline temperature accordingly. Patient and system data are displayed on the system screen or can be synchronized with hospital monitors.
  The working principle of this method [7] involves temperature regulation via a specialized circuit that maintains a constant body temperature or gradually increases the core temperature in a controlled manner, based on the patient’s clinical needs. This temperature management system is typically used in critical cases, such as cardiac arrest, stroke, or traumatic brain injury [8], where precise control of body temperature can be vital for improving clinical outcomes [9]. The key features of this procedures are the following:
  (a) Precise temperature control: The system enables the precise regulation of the patient’s temperature, whether cooling or warming is required. This capability is essential in therapeutic hypothermia or targeted temperature management (TTM).
  (b) Rapid temperature adjustment: The system can quickly adjust a patient’s temperature, which is critical in emergency situations where time is a decisive factor.
  (c) Closed-circuit system: It operates using a closed-loop system with catheters circulating thermally controlled saline, ensuring efficient heat exchange without relying on intravenous fluid administration.
  (d) Unlike other methods, this system does not require extracorporeal circulation.
2. ECMO treatment (Extracorporeal Membrane Oxygenation) in hypothermia
  ECMO is a life-support technique used in cases of severe hypothermia, particularly when conventional rewarming methods fail or when the patient experiences cardiac or respiratory failure.
  ECMO is indicated in severe hypothermia (core temperature typically <28 °C) associated with cardiac or respiratory failure, especially in patients in cardiac arrest or those with refractory hypothermia (unresponsive to conventional rewarming methods such as external rewarming or internal methods like heated fluids).
  The ECMO circuit is initiated by inserting large cannulas (tubes) into major blood vessels. In veno-arterial (VA) ECMO, blood is drained from a large vein (typically the femoral or jugular vein), passed through the ECMO device for oxygenation and warming, and then returned to the body through a major artery (usually the femoral or carotid artery).
  Blood is gradually warmed as it circulates through the ECMO circuit. This controlled rewarming allows for a steady increase in core body temperature, minimizing complications such as “afterdrop” (the return of cold blood from peripheral tissues to the body core, exacerbating hypothermia) and, despite the complications [10] like bleeding, infection, or mechanical failure, with the following benefits:
  (a) Controlled rewarming: ECMO allows for a gradual and precise increase in core body temperature, reducing the risk of thermal shock or complications like arrhythmias, which are common during rapid rewarming.
  (b) Cardiopulmonary support: Since cardiac function is impaired in hypothermia, ECMO provides critical circulatory support.
  (c) Preservation of organ function: By maintaining circulation and oxygenation, ECMO ensures vital organ function is sustained during the rewarming process.

Between IVTM and ECMO, there are notable differences in terms of applicability, target population, cost, availability, and the complications that may result from the use of these rewarming methods (Table 1).

Severe hypothermia is life-threatening, and the clinical presentation of this condition is crucial in selecting the appropriate rewarming strategy. In the case presented, given that the patient was hemodynamically stable, without signs of myocardial ischemia or multi-organ complications, passive rewarming was initially chosen to avoid any potential complications associated with the use of ECMO or IVTM devices. It should be noted that this patient had significant known and unknown risk factors (notably, the patient was therapeutically neglected and lived in severely unsanitary conditions). Since the patient did not respond to passive rewarming therapy, the decision was made to proceed with IVTM, owing to the patient’s maintained hemodynamic stability, the absence of multi-organ dysfunction, and the inability to rule out preexisting pathologies that might be exacerbated by the volume overload associated with ECMO. Furthermore, the greater availability and lower cost of IVTM relative to ECMO, along with limitations in the operational and monitoring infrastructure for patients on ECMO, supported this choice. Ultimately, the decision to actively rewarm the patient using IVTM yielded favorable outcomes, as evidenced by an improvement in the patient’s clinical condition, which prompted the preparation of this case report.

Severe hypothermia can lead to critical complications [11], including the following: cardiac arrhythmias, respiratory failure (central cause), metabolic acidosis, coagulopathies. Severe hypothermia disrupts normal physiological functions at multiple levels [12].

Nonetheless, the patient’s clinical course deteriorated due to the development of bronchopneumonia and subsequent fatality. Among the complications associated with hypothermia—detailed extensively in Table 2—are pneumonia, sepsis resulting from hypothermia-induced immunosuppression, and an increased susceptibility to nosocomial infections. In this patient, the adverse outcome most likely reflects a cumulative effect of delayed complications related to hypothermia, superimposed on a predisposed clinical environment.

## 4. Challenges Encountered in Case Management

1. Initial slow response to rewarming in a bradycardic and hypertensive patient with risks of fluid overload: Severe hypothermia presents significant challenges in elevating core body temperature through conventional methods such as thermal blankets and the administration of warm intravenous fluids. In this case, initial rewarming during the first two hours achieved only a 0.5 °C/hour increase in core temperature. Given the patient’s systolic blood pressure exceeding 160 mmHg, continued intravascular fluid administration posed risks of volume overload, necessitating the adoption of advanced rewarming techniques.
2. Invasiveness of central venous catheter placement for IVTM: The invasive nature of inserting a specialized central venous catheter, typically via a femoral approach, poses additional challenges. The complications of the procedure are operator-dependent and similar to those associated with the insertion of a temporary dialysis catheter via femoral access.
3. Cost [13] of advanced rewarming techniques: Advanced methods for managing severe hypothermia are significantly more expensive [14] compared to conventional active rewarming therapies. However, when comparing the IVTM technique used in this patient to extracorporeal membrane oxygenation (ECMO), there are notable cost differences. These differences may justify the selective use of IVTM, particularly in patients with contraindications to intravascular volume administration.

## 5. Conclusions

This case highlights the critical importance of rapid and effective intervention in managing severe hypothermia, a condition that, without prompt and appropriate treatment, can lead to severe complications and even death. The use of an intravascular warming system allowed for controlled and gradual rewarming, minimizing the risks associated with rapid rewarming, such as arrhythmias and rewarming shock. This approach demonstrated significant benefits in stabilizing vital functions and reducing complications, underscoring the essential role of advanced rewarming technologies in the management of patients with severe hypothermia [15].

For patients with severe hypothermia and restrictions on intravenous fluid administration, we recommend the use of an advanced, non-extracorporeal controlled temperature warming system as the preferred strategy for rewarming, aiming to minimize immediate and medium-term risks.

In our institution’s experience, for patients with severe hypothermia who do not have contraindications to conventional active warming methods, and where continuous monitoring of vital signs and internal temperature is feasible, there is currently insufficient evidence to demonstrate a proven benefit of using such an advanced system compared to conventional warming techniques.

## Figures and Tables

**Table 1:** An illustration of the parallels between IVTM and ECMO, emphasizing the key fundamental elements that guide the decision-making process in selecting one method over the other as an intervetion for correcting hypothermia.

Criteria	Intravascular Temperature Management (IVTM)	Extracorporeal Membrane Oxygenation (ECMO)
Main purpose	Intravascular temperature control for gradual rewarming	Extracorporeal life support and rewarming
Mechanism	Catheter inserted intravascularly to regulate temperature via fluid pefusion or heat exchange	Extracorporeal circuit that oxygenates and warms the blood
Indications	Severe hypothermia without cardiac arrest Hemodynamically stable patients	Severe hypothermia with cardiac arrest Multi-organ dysfunction
Contraindications	Absolute Severe systemic infections Severe thrombocytopenia or clotting disorders Allergy or intolerance to catheter materials Relative Severe hemodynamic instability Vascular obstuctions Extreme hypothermia with cardiac arrest	Absolute Irreversible terminal disease Severe clotting disorders Patients with severe contraindications for aticoagulation Relative Elderly patients Severe chronic conditions Uncontrolled active infections Extreme obesity
Limitations	Catheter-related issues (infection, thrombosis) Limited availability in some hospitals Limited effectiveness in cardiac arrest	Very high costs Requires advanced infrastructure Increased risk of complications (bleeding, infection)
Complications	Insertion site infections Thrombophlebitis Risk of vascular perforation	Systemic infection Severe bleeding Thrombosis in the circuit Mechanical device complications
Ideal cases of application	Isolated hypothermia without cardiac arrest Patients at risk of fluid overload	Hypothermia with cardiac arrest Severe organ dysfunction or urgent oxygenation needs
Availability	More commonly available in general hospitals	Available only in specialized centers and large hospitals
Costs	Significantly lower than ECMO	Extremely high, including equipment, personnel, consumables
Associated survival rates	Insufficient data to confirm	Associated with higher survival rates in severe cases

**Table 2:** The presentation of both immediate and delayed complications associated with the use of IVTM and ECMO for correcting hypothermia serves not only to guide the tailoring of rewarming therapy according to the patient’s risk factors but also as aiding in the therapeutic evaluation and the differential diagnosis following the rewarming process, taking into account the long-term risks associated with these procedures.

Nr. Crt.	Immediate Complications	Delayed Complications
Intravascular Temperature Management (IVTM)	Cardiac arrhythmias (ventricular fibrillation, ventricular tachycardia, bradycardia) Rewarming shock with severe hypotension Myocardial ischemia Rewarming syndrome: metabolic acidosis, hyperkalimia, hypoglycemia Cerebral edema Seizures Pulmonary edema: non-cardiogenic induced by rewarming; or cardiogenic secondary to heart failure Hypoventilation Acute renal failure associated with severe hypotension or rhabdomuolysis Coagulopathy-DIC Thrombembolism	Recurence of hypothermia Paradoxical hyperthermia—in aggresive rewarming Secondary infections/Nosocomial infections Multiple Organ Dysfunction Syndrome—due to reperfusion injury Persistent neurological symptoms Residual metabolic disorders: hypokalemia, hypomagnesemia, metabolic acidosis Post-hypothermia syndrome—chronic fatigue, confusion, anxiety, depression
Extracorporeal Membrane Oxygenation (ECMO)	Cardiac arrhythmias (ventricular fibrillation, ventricular tachycardia, bradycardia) Myocardial ischemia Arterial hypotension Pulmonary edema Atelectasis Cerebral edema Seizures Persistent coma Bleeding Thrombocytopenia Metabolic complications: hyperkalemia, metabolic acidosis Arterial or venous dissection Air embolism or thrombosis	Recurence of hypothermia Pneumonia Pulmonary fibrosis—prolonged inflammation or aggresive mechanical ventilation Catheter infection DIC Hemolytic anemia—due to cellular trauma related to blood passage through the ECMO Cognitive or motor deficits Stroke Chronic renal failure Post-intensive care syndrome Delayed rehabilitation

## Data Availability

There is no data associated with this manuscript.
